# Supplementary material for: Machine Learning-Based Modeling of Olfactory Receptors in Their Inactive State: Human OR51E2 as a Case Study
Source: J Chem Inf Model. 2023 May 5;63(10):2911–7. doi: 10.1021/acs.jcim.3c00380 (PMC10207261; doi:10.1021/acs.jcim.3c00380)
Supplement: Supplementary file 1 — ci3c00380_si_001.pdf [file ci3c00380_si_001.pdf]

Supporting Information: Machine Learning-based Modeling of Olfactory Receptors in their Inactive State: Human OR51E2 as a Case Study

Mercedes Alfonso-Prieto*,† and Riccardo Capelli*,‡

†*Computational Biomedicine, Institute for Advanced Simulation IAS-5/Institute for Neuroscience and Medicine INM-9, Forschungszentrum Jülich GmbH, Wilhelm-Johnen-Straße, D-52428 Jülich, Germany*

‡*Dipartimento di Bioscienze, Università degli Studi di Milano, Via Celoria 26, I-20133 Milan, Italy*

E-mail: m.alfonso-prieto@fz-juelich.de; ricccardo.capelli@unimi.it

Initial structures generation

The hOR51E2 sequence was taken from UniProt[1] (accession code: Q9H255, amino acids 1-320). Such sequence was employed as input for all the structure prediction algorithms listed below.

AlphaFold (AF). We obtained the initial coordinates (accession code AF-Q9H255-F1, v3) from the AlphaFold Protein Structure Database[2] (https://alphafold.ebi.ac.uk); such model was generated with the vanilla version of Alphafold.[3]

AlphaFold, inactive state ($\mathbf{AF_{in}}$). AlphaFold weights were trained on all experimental structures available at the PDB, regardless of their conformation in the case of multi-state proteins. Heo and Feig recently proposed[4] to perform structure predictions considering mul-

tiple sequence alignments built on state-annotated data (*i.e.,* using only active or inactive conformations for GPCRs). Since our intention was to model the apo receptor (*i.e.,* without ligands), we therefore chose the inactive conformation. In practice, such model was obtained from GPCRdb[5] (https://gpcrdb.org), which maintains an updated database of GPCR models generated with AlphaFold-MultiState.

RoseTTAFold (RF). The structure predicted by RoseTTAFold[6] was obtained via the web server of the Baker Lab, ROBETTA (https://robetta.bakerlab.org).

OmegaFold (OF). The OmegaFold[7] prediction was run on local resources using the public implementation available on GitHub (https://github.com/HeliXonProtein/OmegaFold), release v.1.1.0, with default parameters.

ESMFold (EF). The structure from ESMFold[8] was obtained from the python notebook available through the ColabFold interface (https://github.com/sokrypton/ColabFold).

SwissModel (SM). A homology model was retrieved from the SwissModel repository.[9] The template used was the human neuropeptide Y receptor type 2 (PDB structure 7X9B, chain D), which has a sequence identity of 18.12% with hOR51E2. Unfortunately, hORs exhibit a low sequence identity ($<$ 20%) with other class A GPCRs and thus a better template cannot be found.[10] The SM model covers residues 22-307.

System preparation

Initially, we preprocessed all the structures obtained via *ab initio* and homology modeling algorithms by using the Protein Preparation Wizard implemented in Schrödinger Maestro version 2022-3,[11] which automatically assigns amino acid protonation states based on their microenvironment. Two exceptions were represented by D69$^{2.50}$ and E110$^{3.39}$, that were kept in the charged state. All the structures prepared in this way were further processed via the online interface of CHARMM-GUI[12,13] (https://charmm-gui.org/). First, we built a disulphide bond between C96$^{3.25}$ and C178$^{45.50}$. Then, we defined a cubic simulation box

with dimensions (100 Å) × (100 Å) × (120 Å), with the receptor in the center, embedded in a POPC membrane. The lipid bilayer and the receptor were solvated in water with a NaCl concentration of 150 mM, in line with standard experimental and physiological conditions for GPCRs. The protein, lipids, and ions were parameterized using the CHARMM36m force field,[14] while water was described with the TIP3P[15] model.

Molecular dynamics simulations

Our protocol is a modified and extended version of the standard CHARMM-GUI-suggested workflow for equilibration and production runs of transmembrane proteins. It comprises nine different steps (here we consider the same numbering used in the CHARMM-GUI input files, 0-6, plus two additional steps 7-8):

- step 0: 5,000 steps of steepest descent minimization, restraining the protein backbone ($k = 4,000$ kJ/mol/nm^2) and sidechains ($k = 2,000$ kJ/mol/nm^2), lipid phosphate groups ($k = 1,000$ kJ/mol/nm^2) and dihedrals ($k = 1,000$ kJ/mol/rad^2).
- step 1: 125 ps of MD with a time step of 1 fs, restraining the protein backbone ($k = 4,000$ kJ/mol/nm^2) and sidechains ($k = 2,000$ kJ/mol/nm^2), lipid phosphate groups ($k = 1,000$ kJ/mol/nm^2) and dihedrals ($k = 1,000$ kJ/mol/rad^2).
- step 2: 125 ps of MD with a time step of 1 fs, restraining the protein backbone ($k = 2,000$ kJ/mol/nm^2) and sidechains ($k = 1,000$ kJ/mol/nm^2), lipid phosphate groups ($k = 400$ kJ/mol/nm^2) and dihedrals ($k = 400$ kJ/mol/rad^2).
- step 3: 125 ps of MD with a time step of 1 fs, restraining the protein backbone ($k = 1,000$ kJ/mol/nm^2) and sidechains ($k = 500$ kJ/mol/nm^2), lipid phosphate groups ($k = 400$ kJ/mol/nm^2) and dihedrals ($k = 200$ kJ/mol/rad^2).
- step 4: 500 ps of MD with a time step of 2 fs, restraining the protein backbone ($k = 500$ kJ/mol/nm^2) and sidechains ($k = 200$ kJ/mol/nm^2), lipid phosphate groups ($k = 200$

kJ/mol/nm^2) and dihedrals ($k = 200$ kJ/mol/rad^2).

- step 5: 500 ps of MD with a time step of 2 fs, restraining the protein backbone ($k = 200$ kJ/mol/nm^2) and sidechains ($k = 50$ kJ/mol/nm^2), lipid phosphate groups ($k = 40$ kJ/mol/nm^2) and dihedrals ($k = 100$ kJ/mol/rad^2).

- step 6: 100 ns of MD with a time step of 2 fs, restraining the protein backbone ($k = 50$ kJ/mol/nm^2); this step is 10-fold longer than in the standard CHARMM-GUI protocol.

- step 7: 100 ns of MD with a time step of 2 fs, restraining the protein backbone ($k = 5$ kJ/mol/nm^2); this is a completely new step, which increases the length of the restrained equilibration.

The last, production step (8) consists of $2.5 \cdot 10^8$ steps with a time step of 2 fs (500 ns) of unrestrained MD simulation. For both van der Waals and short-range interactions, a cutoff distance was set at 10 Å and long-range electrostatic interactions were computed via the smooth particle mesh Ewald summation method.[16] Temperature was kept at 310 K via velocity rescale thermostat[17] with a coupling time of 0.2 ps on three groups: protein, membrane, and water and ions. Pressure was kept constant at 1 bar with the semi-isotropic cell rescale barostat[18] with a coupling time of 0.5 ps. All the simulations were performed using GROMACS[19] version 2021.2; three replicas were run for each model.

A^{100} activation index

A^{100} is an estimator of the activation state of class A GPCR structures,[20] defined as a weighted sum of distances between C_α atoms of selected residues, namely:

$$\begin{aligned} A^{100} = & -14.43 \cdot d(V^{1.53}, L^{7.55}) - 7.62 \cdot d(D^{2.50}, T^{3.37}) + \\ & + 9.11 \cdot d(N^{3.42}, I^{4.42}) - 6.32 \cdot d(W^{5.66}, A^{6.34}) + \\ & - 5.22 \cdot d(L^{6.58}, Y^{7.35}) + 278.88 \end{aligned} \tag{1}$$

where $d(X, Y)$ is the Euclidean distance between residues X and Y. In equation (1) the amino acid names are based on the generic residue numbering proposed by Ballesteros and Weinstein[21] (BW) for class A GPCRs. In the case of hOR51E2, the BW numbering was taken from GPCRdb[5] (see Table S1). We would like to note here that, because of the lack of conservation of some class A GPCR motifs in ORs (see Table S3), the nature of some residues is different, yet the BW position is the same. Thus, the A^{100} activation index for hOR51E2 is defined as:

$$\begin{aligned} A^{100}_{OR51E2} = & -14.43 \cdot d(V44^{1.53}, A293^{7.55}) - 7.62 \cdot d(D69^{2.50}, A108^{3.37}) + \\ & + 9.11 \cdot d(I113^{3.42}, T140^{4.42}) - 6.32 \cdot d(L225^{5.66}, F237^{6.34}) + \\ & - 5.22 \cdot d(H261^{6.58}, V274^{7.35}) + 278.88 \end{aligned} \tag{2}$$

This estimator has been implemented in PLUMED 2.8.[22,23]

As shown in Figure S1, the A^{100} index remains around a value of 11 throughout the entire 500 ns simulation for all six systems, regardless of the replica considered. Following the two-state model of the original work,[20] where a class A GPCR structure is defined as in the inactive state for an index value smaller than 30, we can consider all the simulated models in the inactive state.

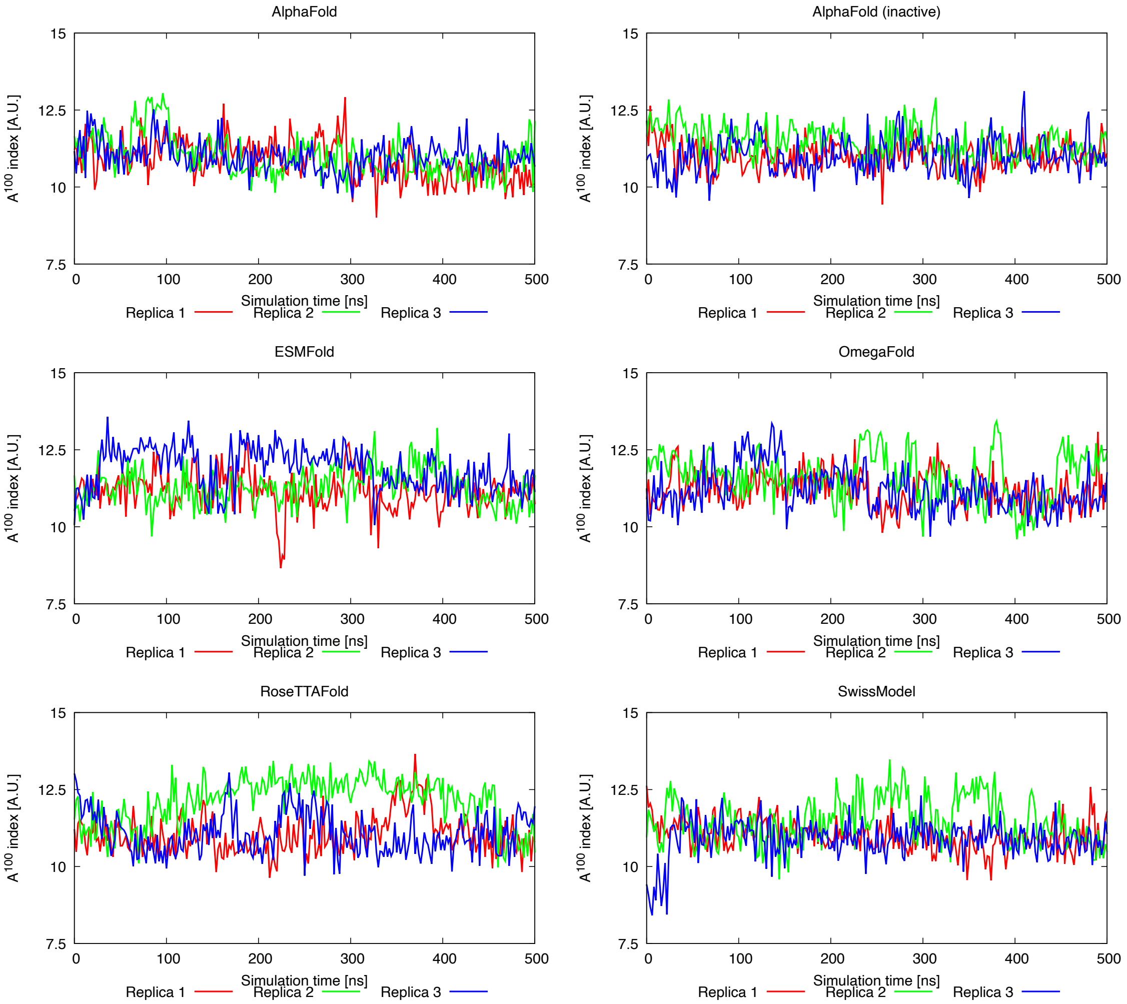

Figure S1: Time evolution of the A^{100} activation index for the eighteen MD simulations of sodium-bound hOR51E2 performed in this work.

A^{100} implementation in PLUMED for hOR51E2

```
UNITS LENGTH=A

WHOLEMOLECULES ENTITY0=1-4571


# We are here considering the C-alpha distances suggested in Ibrahim et al. JCIM 2019

# the numbering refers to hOR51E2

d1: DISTANCE ATOMS=340,4325

d2: DISTANCE ATOMS=757,1381

d3: DISTANCE ATOMS=1450,1881

d4: DISTANCE ATOMS=3246,3440

d5: DISTANCE ATOMS=3801,4019


# And this is the eq.1 of the same work cited above

MATHEVAL ...

  ARG=d1,d2,d3,d4,d5

  VAR=x1,x2,x3,x4,x5

  FUNC=-14.43*x1-7.62*x2+9.11*x3-6.32*x4-5.22*x5+278.88

  LABEL=a100

  PERIODIC=NO

... MATHEVAL


PRINT FILE=a100.dat ARG=d1,d2,d3,d4,d5,a100 STRIDE=1


ENDPLUMED
```

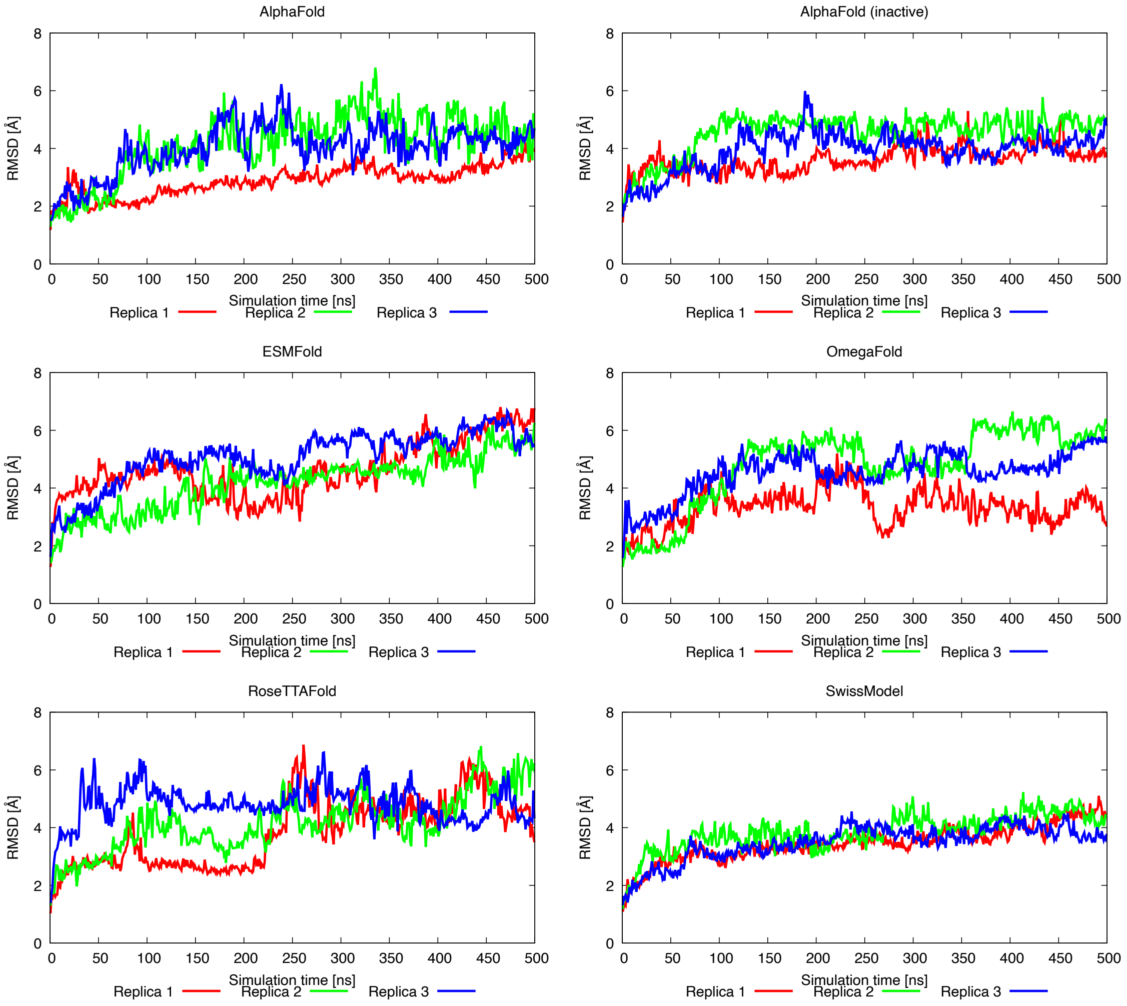

Figure S2: Time evolution of the RMSD of the heavy atoms of the whole protein for the eighteen MD simulations of sodium-bound hOR51E2 performed in this work.

Table S1: Ballesteros-Weinstein generic numbering for hOR51E2, as listed in the GPCRdb[5] (https://gpcrdb.org/residue/residuetabledisplay, accessed on March 2023).

**TM1**		**TM2**		**TM3**		**TM4**		**TM5**		**TM6**		**TM7**	
1x32	H23	2x37	A56	3x21	S92	4x38	N136	5x32	T191	6x26	S229	7x29	H268
1x33	F24	2x38	P57	3x22	F93	4x39	N137	5x33	L192	6x27	K230	7x30	P269
1x34	W25	2x39	M58	3x23	E94	4x40	T138	5x34	P193	6x28	S231	7x31	I270
1x35	V26	2x40	Y59	3x24	A95	4x41	V139	5x35	N194	6x29	E232	7x32	V271
1x36	G27	2x41	L60	3x25	C96	4x42	T140	5x36	V195	6x30	R233	7x33	R272
1x37	F28	2x42	F61	3x26	L97	4x43	A141	5x37	V196	6x31	A234	7x34	V273
1x38	P29	2x43	L62	3x27	T98	4x44	Q142	5x38	Y197	6x32	K235	7x35	V274
1x39	L30	2x44	C63	3x28	Q99	4x45	I143	5x39	G198	6x33	A236	7x36	M275
1x40	L31	2x45	M64	3x29	M100	4x46	G144	5x40	L199	6x34	F237	7x37	G276
1x41	S32	2x46	L65	3x30	F101	4x47	I145	5x41	T200	6x35	G238	7x38	D277
1x42	M33	2x47	A66	3x31	F102	4x48	V146	5x42	A201	6x36	T239	7x39	I278
1x43	Y34	2x48	A67	3x32	I103	4x49	A147	5x43	I202	6x37	C240	7x40	Y279
1x44	V35	2x49	I68	3x33	H104	4x50	V148	5x44	L203	6x38	V241	7x41	L280
1x45	V36	2x50	D69	3x34	A105	4x51	V149	5x45	L204	6x39	S242	7x42	L281
1x46	A37	2x51	L70	3x35	L106	4x52	R150	5x46	V205	6x40	H243	7x43	L282
1x47	M38	2x52	A71	3x36	S107	4x53	G151	5x47	M206	6x41	I244	7x45	P283
1x48	F39	2x53	L72	3x37	A108	4x54	S152	5x48	G207	6x42	G245	7x46	P284
1x49	G40	2x54	S73	3x38	I109	4x55	L153	5x49	V208	6x43	V246	7x47	V285
1x50	N41	2x55	T74	3x39	E110	4x56	F154	5x50	D209	6x44	V247	7x48	I286
1x51	C42	2x551	S75	3x40	S111	4x57	F155	5x51	V210	6x45	L248	7x49	N287
1x52	I43	2x56	T76	3x41	T112	4x58	F156	5x52	M211	6x46	A249	7x50	P288
1x53	V44	2x57	M77	3x42	I113	4x59	P157	5x53	F212	6x47	F250	7x51	I289
1x54	V45	2x58	P78	3x43	L114	4x60	L158	5x54	I213	6x48	Y251	7x52	I290
1x55	F46	2x59	K79	3x44	L115	4x61	P159	5x55	S214	6x49	V252	7x53	Y291
1x56	I47	2x60	I80	3x45	A116	4x62	L160	5x56	L215	6x50	P253	7x54	G292
1x57	V48	2x61	L81	3x46	M117	4x63	L161	5x57	S216	6x51	L254	7x55	A293
1x58	R49	2x62	A82	3x47	A118	4x64	I162	5x58	Y217	6x52	I255	7x56	K294
1x59	T50	2x63	L83	3x48	F119	4x65	K163	5x59	F218	6x53	G256		
1x60	E51	2x64	F84	3x49	D120	4x66	R164	5x60	L219	6x54	L257		
		2x65	W85	3x50	R121	4x67	L165	5x61	I220	6x55	S258		
		2x66	F86	3x51	Y122			5x62	I221	6x56	V259		
		2x67	D87	3x52	V123			5x63	R222	6x57	V260		
				3x53	A124			5x64	T223	6x58	H261		
				3x54	I125			5x65	V224	6x59	R262		
				3x55	C126			5x66	L225	6x60	F263		
				3x56	H127			5x67	Q226	6x61	G264		
								5x68	L227				

**ICL1**				**ICL2**		**ECL2**						**H8**	
12x48	R52			34x50	P128	45x50	C178					8x47	T295
12x49	S53			34x51	L129	45x51	V179					8x48	K296
12x50	L54			34x52	R130	45x52	H180					8x49	Q297
12x51	H55			34x53	H131							8x50	I298
				34x54	A132							8x51	R299
				34x55	A133							8x52	T300
				34x56	V134							8x53	R301
				34x57	L135							8x54	V302
												8x55	L303
												8x56	A304
												8x57	M305
												8x58	F306
												8x59	K307
												8x60	I308

Table S2: Water passage observed in MD simulations.

Predictor	Replica 1	Replica 2	Replica 3
AF	Yes	**No**	**No**
AF_{in}	**No**	**No**	**No**
EF	Yes	Yes	Yes
OF	**No**	Yes	Yes
RF	Yes	Yes	Yes
SM	Yes	Yes	Yes

Comparison of the *in silico* models with sodium ion bound

We assessed the similarity of the conformations explored during the MD simulations by calculating the mutual backbone RMSD and using Multidimensional Scaling (MDS), a non-linear dimensionality reduction approach to visualize the information contained in the RMSD matrix into a 2D projection,[24] shown in Figure S3. We qualitatively confirmed the same results as in Figure 1: AF and OF are superimposed, while all the other models are well-separated.

In addition to clustering analysis reported in the main text, we also performed a structure-based sequence alignment of the cluster centroid structures shown in Figure 3, using the STAMP algorithm implemented in the MultiSeq tool[25] of VMD[26] (version 1.9.4a57). Such alignment (Figure S4) reveals that TM6 contains the largest number of mismatches within the helix, followed by TM7. This is in line with the differences in the TM6-TM7 interface described in the main text (see Figure 4).

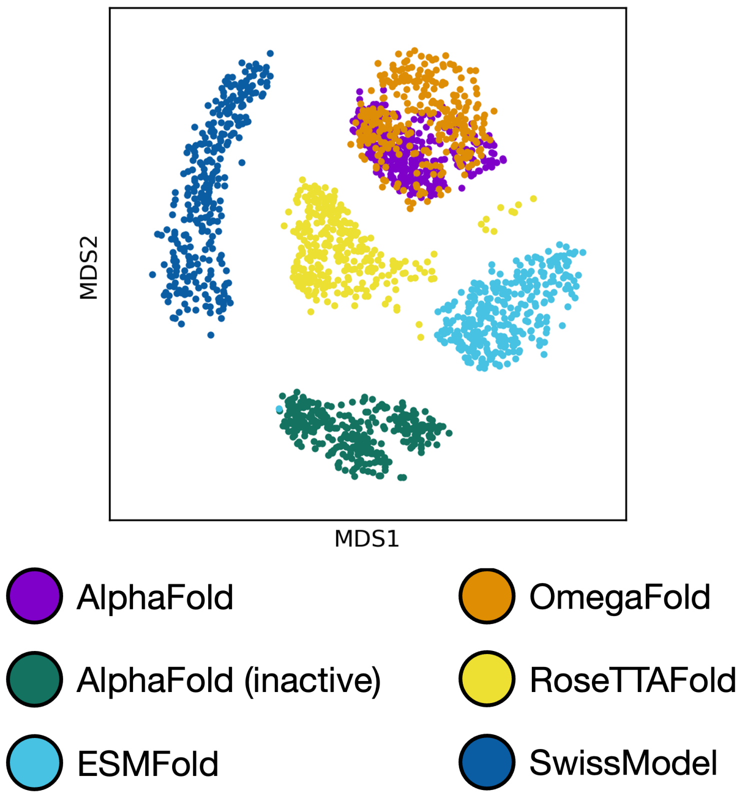

Figure S3: MDS-based similarity representation of all the sodium-bound hOR51E2 trajectories.

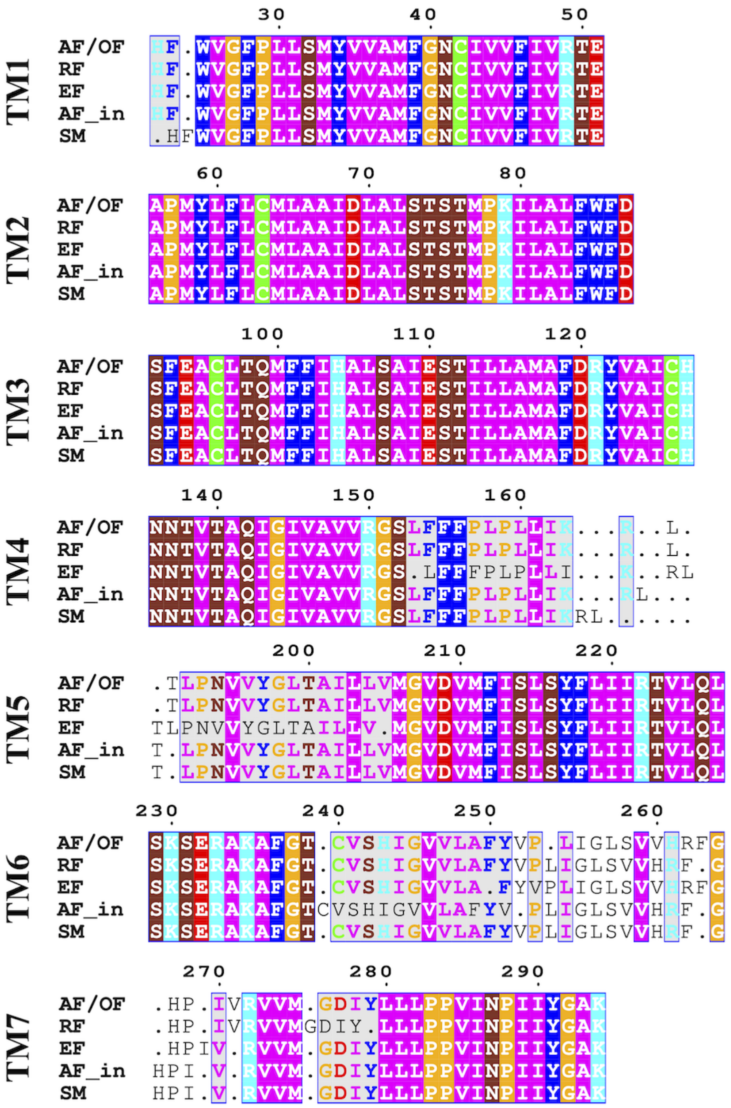

Figure S4: Structure-based multiple sequence alignment of the cluster centroid structures shown in Figure 3. The MSA was visualized using ESPript tool.[27] Residues identically aligned in all six models are displayed with white characters and enclosed in a box colored according to their physicochemical properties, residues showing a mismatch in one model are shown within a grey box and font color based on their properties, and gaps and residues mismatched in two or more models are shown in black font.

Comparison of the two best *in silico* models with the experimental structure of hOR51E2

We used the cryo-EM structure of hOR51E2 in the active state (PDB 8F76) to validate the two best inactive models according to our MD-based protocol, AF/OF and AF_{in} (i.e. the centroid structures of clusters 1 and 4 in Figure 2). We first calculated the backbone RMSD of the seven transmembrane (TM) helical bundle, using the definition of the TM helices listed in Table S1 and the experimental structure as reference. The obtained RMSD values are 2.46 Å and 3.54 Å for the AF/OF and AF_{in} models, respectively. However, such overall RMSD reflects both structural differences due to the quality of the models and conformational changes upon receptor activation. Indeed, the experimental structure was solved in the active state, whereas the computational models correspond to distinct inactive states (see Figure S1). Hence, we additionally quantified the structural changes due to the different activation state of the three structures using the 7x7 RMSD matrix of the individual TM helices, as defined in reference.[28] Such matrix was obtained with VMD[26] (version 1.9.4a57) by successively fitting one of the seven TM helices to the reference structure (here PDB 8F76) and calculating the backbone RMSD for each of the seven TM helices; for further details see the 7x7_rmsd.tcl script available in the zenodo repository (https://doi.org/10.5281/zenodo.7817679). The corresponding matrices are shown in Figure S5; each row represents the superposition to helix i (S-TMi) and each column the backbone RMSD of helix j of the model with respect to the same helix in the experimental structure (TMj:TMj). The TM helix showing the largest backbone RMSD is TM6, in line with conformational changes in this helix being necessary for activation of hOR51E2[29] in particular and class A GPCRs in general.[30] In this regard, it is worth noting that the AF/OF models were trained on both active and inactive GPCR experimental structures, whereas AF_{in} only on inactive GPCR structures.

In addition, we analyzed (i) the residue-residue contacts at the interface between TM6 and TM7 and (ii) the side chain orientation of the residue lining the ligand binding site based

on the cryo-EM structure. Such comparisons are reported in the main text and revealed a better qualitative agreement of the AF_{in} model with the experimental structure.

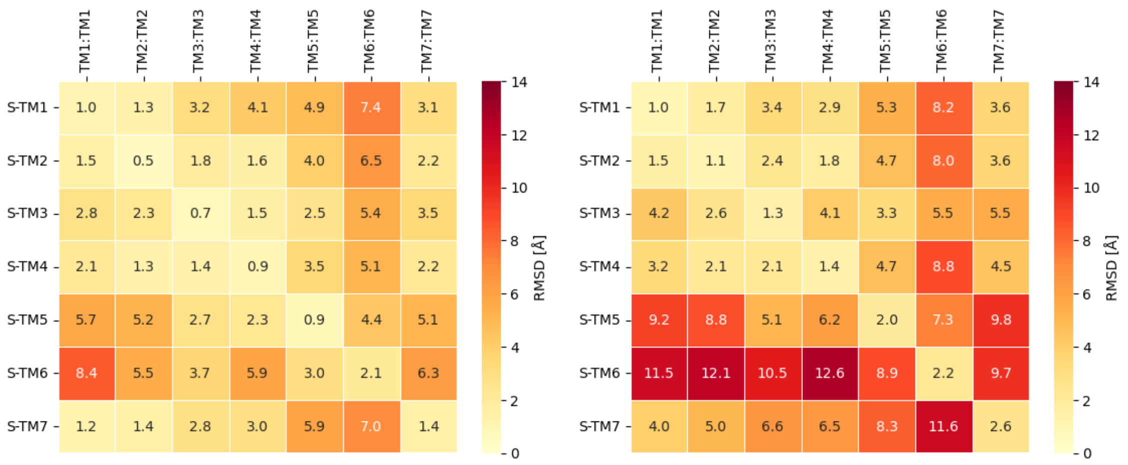

Figure S5: 7x7 RMSD calculation of AF/OF (Left) and AF_{in} (Right) cluster centroids vs. the experimental hOR51E2 cryoEM structure (PDB 8F76).

Table S3: Conserved motifs in hORs with respect to class A GPCRs. Data taken from references[10,31–34]

TM helix	hORs	class A GPCRs
TM1	$G^{1.49}N^{1.50}xxI^{1.53}$	$xN^{1.50}xxV^{1.53}$
TM2	$L^{2.46}S^{2.47}xxD^{2.50}$	$L^{2.46}xxxD^{2.50}$
TM3	$D[E]^{3.39}$	$S^{3.39}$
	$M^{3.46}A^{3.47}Y[F]^{3.48}$	–
	$D^{3.49}R^{3.50}Y^{3.51}$	$D[E]^{3.49}R^{3.50}Y^{3.51}$
TM4	$W^{4.50}$	$W^{4.50}$
TM5	–	$P^{5.50}$
	$S^{5.57}Y^{5.58}$	–
TM6	$KAFSTCxSH^{6.40}$	–
	$V[I]^{6.44}xxF[Y]^{6.47}Y[F]^{6.48}$	$F^{6.44}xxxW^{6.48}$
	$G^{6.49}x$	$P^{6.50}$
TM7	$N^{7.49}P7.50xI[L]^{7.52}Y^{7.53}$	$N^{7.49}P^{7.50}xxY^{7.53}$

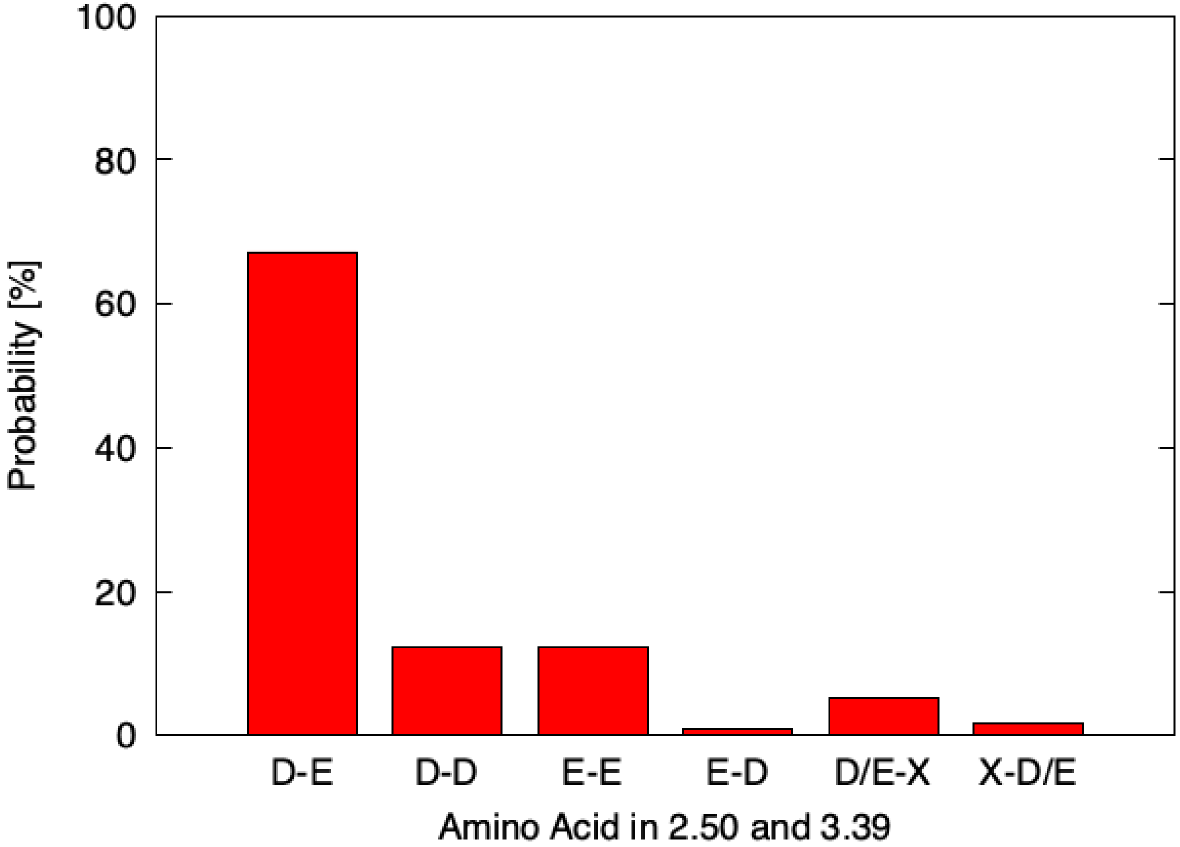

Figure S6: Residue conservation in BW positions 2.50 and 3.39 across human olfactory receptors, based on the MSA of 412 hORs in reference.[10]

Table S4: Residue conservation of the sodium pocket in hORs and class A GPCRs. The fifteen positions included were selected based on reference.[35] Data for hORs was taken from the MSA of 412 hORs in reference,[10] whereas for class A GPCRs it was aggregated from the 259 receptors analyzed in reference;[35] conservation frequency is indicated between parentheses as percentage. The two positions discussed in the main text (2.50 and 3.39) are highlighted in red.

**Position**	**hORs**	**class A GPCRs**
1.50	N (99)	N (98)
1.53	I (62)	V (67)
2.46	L (93)	L (91)
2.47	S (74) / A (24)	A (75)
2.49	L (31) / A (19)	A (59) / S (24)
2.50	D (82) / E (16)	D (94)
3.35	F (43) / L (39)	S (20) / N (29)
3.39	E (81) / D (13)	S (76)
3.43	L (93)	L (76)
6.44	V (83)	F (79)
6.48	Y (68) / F (24)	W (67) / F (15)
7.45	T (50) / P (17) / I (15)	N (70)
7.46	P (96)	S (64)
7.49	N (98)	N (74) / D (20)
7.50	P (97)	P (94)
7.53	Y (96)	Y (91)

References

(1) The UniProt Consortium, UniProt: the universal protein knowledgebase in 2021. *Nucleic Acids Research* **2020**, *49*, D480–D489.

(2) Varadi, M.; Anyango, S.; Deshpande, M.; Nair, S.; Natassia, C.; Yordanova, G.; Yuan, D.; Stroe, O.; Wood, G.; Laydon, A.; Žídek, A.; Green, T.; Tunyasuvunakool, K.; Petersen, S.; Jumper, J.; Clancy, E.; Green, R.; Vora, A.; Lutfi, M.; Figurnov, M.; Cowie, A.; Hobbs, N.; Kohli, P.; Kleywegt, G.; Birney, E.; Hassabis, D.; Velankar, S. AlphaFold Protein Structure Database: massively expanding the structural coverage of protein-sequence space with high-accuracy models. *Nucleic Acids Research* **2021**, *50*, D439–D444.

(3) Jumper, J.; Evans, R.; Pritzel, A.; Green, T.; Figurnov, M.; Ronneberger, O.; Tunyasuvunakool, K.; Bates, R.; Žídek, A.; Potapenko, A.; Bridgland, A.; Meyer, C.; Kohl, S. A. A.; Ballard, A. J.; Cowie, A.; Romera-Paredes, B.; Nikolov, S.; Jain, R.; Adler, J.; Back, T.; Petersen, S.; Reiman, D.; Clancy, E.; Zielinski, M.; Steinegger, M.; Pacholska, M.; Berghammer, T.; Bodenstein, S.; Silver, D.; Vinyals, O.; Senior, A. W.; Kavukcuoglu, K.; Kohli, P.; Hassabis, D. Highly accurate protein structure prediction with AlphaFold. *Nature* **2021**, *596*, 583–589.

(4) Heo, L.; Feig, M. Multi-State modeling of G-protein coupled receptors at experimental accuracy. *Proteins: Structure, Function, and Bioinformatics* **2022**,

(5) Pándy-Szekeres, G.; Caroli, J.; Mamyrbekov, A.; Kermani, A. A.; Keserű, G. M.; Kooistra, A. J.; Gloriam, D. E. GPCRdb in 2023: state-specific structure models using AlphaFold2 and new ligand resources. *Nucleic Acids Research* **2022**,

(6) Baek, M.; DiMaio, F.; Anishchenko, I.; Dauparas, J.; Ovchinnikov, S.; Lee, G. R.; Wang, J.; Cong, Q.; Kinch, L. N.; Schaeffer, R. D.; Claudia, M.; Park, H.; Adams, C.; Glassman, C. R.; DeGiovanni, A.; Pereira, J. H.; Rodrigues, A. V.; van Dijk, A. A.;

Ebrecht, A. C.; Opperman, D. J.; Sagmeister, T.; Buhlheller, C.; Pavkov-Keller, T.; Rathinaswamy, M. K.; Dalwadi, U.; Yip, C. K.; Burke, J. E.; Garcia, K. C.; Grishin, N. V.; Adams, P. D.; Read, R. J.; Baker, D. Accurate prediction of protein structures and interactions using a three-track neural network. *Science* **2021**, *373*, 871–876.

(7) Wu, R.; Ding, F.; Wang, R.; Shen, R.; Zhang, X.; Luo, S.; Su, C.; Wu, Z.; Xie, Q.; Berger, B.; Ma, J.; Peng, J. High-resolution de novo structure prediction from primary sequence. *bioRxiv* **2022**,

(8) Lin, Z.; Akin, H.; Rao, R.; Hie, B.; Zhu, Z.; Lu, W.; Smetanin, N.; Verkuil, R.; Kabeli, O.; Shmueli, Y.; dos Santos Costa, A.; Fazel-Zarandi, M.; Sercu, T.; Candido, S.; Rives, A. Evolutionary-scale prediction of atomic-level protein structure with a language model. *Science* **2023**, *379*, 1123–1130.

(9) Waterhouse, A.; Bertoni, M.; Bienert, S.; Studer, G.; Tauriello, G.; Gumienny, R.; Heer, F. T.; de Beer, T. A.; Rempfer, C.; Bordoli, L.; Lepore, R.; Schwede, T. SWISS-MODEL: homology modelling of protein structures and complexes. *Nucleic Acids Research* **2018**, *46*, W296–W303.

(10) Fierro, F.; Suku, E.; Alfonso-Prieto, M.; Giorgetti, A.; Cichon, S.; Carloni, P. Agonist binding to chemosensory receptors: a systematic bioinformatics analysis. *Frontiers in Molecular Biosciences* **2017**, *4*, 63.

(11) Schrödinger, LLC, Maestro 2022-4, 2022; New York, NY.

(12) Jo, S.; Kim, T.; Iyer, V. G.; Im, W. CHARMM-GUI: a web-based graphical user interface for CHARMM. *Journal of Computational Chemistry* **2008**, *29*, 1859–1865.

(13) Lee, J.; Cheng, X.; Swails, J. M.; Yeom, M. S.; Eastman, P. K.; Lemkul, J. A.; Wei, S.; Buckner, J.; Jeong, J. C.; Qi, Y.; Jo, S.; Pande, V. S.; Case, D. A.; Brooks, C.

L. I.; MacKerell, A. D. J.; Klauda, J. B.; Im, W. CHARMM-GUI input generator for NAMD, GROMACS, AMBER, OpenMM, and CHARMM/OpenMM simulations using the CHARMM36 additive force field. *Journal of Chemical Theory and Computation* **2016**, *12*, 405–413.

(14) Huang, J.; Rauscher, S.; Nawrocki, G.; Ran, T.; Feig, M.; De Groot, B. L.; Grubmüller, H.; MacKerell, A. D. CHARMM36m: an improved force field for folded and intrinsically disordered proteins. *Nature Methods* **2017**, *14*, 71–73.

(15) Jorgensen, W. L.; Chandrasekhar, J.; Madura, J. D.; Impey, R. W.; Klein, M. L. Comparison of simple potential functions for simulating liquid water. *The Journal of Chemical Physics* **1983**, *79*, 926–935.

(16) Essmann, U.; Perera, L.; Berkowitz, M. L.; Darden, T.; Lee, H.; Pedersen, L. G. A smooth particle mesh Ewald method. *The Journal of Chemical Physics* **1995**, *103*, 8577–8593.

(17) Bussi, G.; Donadio, D.; Parrinello, M. Canonical sampling through velocity rescaling. *The Journal of Chemical Physics* **2007**, *126*, 014101.

(18) Bernetti, M.; Bussi, G. Pressure control using stochastic cell rescaling. *The Journal of Chemical Physics* **2020**, *153*, 114107.

(19) Abraham, M. J.; Murtola, T.; Schulz, R.; Páll, S.; Smith, J. C.; Hess, B.; Lindahl, E. GROMACS: High performance molecular simulations through multi-level parallelism from laptops to supercomputers. *SoftwareX* **2015**, *1*, 19–25.

(20) Ibrahim, P.; Wifling, D.; Clark, T. Universal activation index for class A GPCRs. *Journal of Chemical Information and Modeling* **2019**, *59*, 3938–3945.

(21) Ballesteros, J. A.; Weinstein, H. *Methods in neurosciences*; Elsevier, 1995; Vol. 25; pp 366–428.

(22) Tribello, G. A.; Bonomi, M.; Branduardi, D.; Camilloni, C.; Bussi, G. PLUMED 2: New feathers for an old bird. *Computer Physics Communications* **2014**, *185*, 604–613.

(23) Bonomi, M.; Bussi, G.; Camilloni, C.; Tribello, G. A.; Banáš, P.; Barducci, A.; Bernetti, M.; Bolhuis, P. G.; Bottaro, S.; Branduardi, D.; Capelli, R.; Carloni, P.; Ceriotti, M.; Cesari, A.; Chen, H.; Chen, W.; Colizzi, F.; De, S.; De La Pierre, M.; Donadio, D.; Drobot, V.; Ensing, B.; Ferguson, A. L.; Filizola, M.; Fraser, J. S.; Fu, H.; Gasparotto, P.; Gervasio, F. L.; Giberti, F.; Gil-Ley, A.; Giorgino, T.; Heller, G. T.; Hocky, G. M.; Iannuzzi, M.; Invernizzi, M.; Jelfs, K. E.; Jussupow, A.; Kirilin, E.; Laio, A.; Limongelli, V.; Lindorff-Larsen, K.; Löhr, T.; Marinelli, F.; Martin-Samos, L.; Masetti, M.; Meyer, R.; Michaelides, A.; Molteni, C.; Morishita, T.; Nava, M.; Paissoni, C.; Papaleo, E.; Parrinello, M.; Pfaendtner, J.; Piaggi, P. M.; Piccini, G.; Pietropaolo, A.; Pietrucci, F.; Pipolo, S.; Provasi, D.; Quigley, D.; Raiteri, P.; Raniolo, S.; Rydzewski, J.; Salvalaglio, M.; Sosso, G. C.; Spiwok, V.; Šponer, J.; Swenson, D. W. H.; Tiwary, P.; Valsson, O.; Vendruscolo, M.; Voth, G. A.; White, A. Promoting transparency and reproducibility in enhanced molecular simulations. *Nature Methods* **2019**, *16*, 670–673.

(24) *Modern Multidimensional Scaling*; Springer New York, 2005.

(25) Roberts, E.; Eargle, J.; Wright, D.; Luthey-Schulten, Z. MultiSeq: unifying sequence and structure data for evolutionary analysis. *BMC Bioinformatics* **2006**, *7*, 382.

(26) Humphrey, W.; Dalke, A.; Schulten, K. VMD: Visual molecular dynamics. *Journal of Molecular Graphics* **1996**, *14*, 33–38.

(27) Robert, X.; Gouet, P. Deciphering key features in protein structures with the new ENDscript server. *Nucleic Acids Research* **2014**, *42*, W320–W324.

(28) Wang, T.; Wang, Y.; Tang, L.; Duan, Y.; Liu, H. 7 × 7 RMSD matrix: A new method

for quantitative comparison of the transmembrane domain structures in the G-protein coupled receptors. *Journal of Structural Biology* **2017**, *199*, 87–101.

(29) Billesbølle, C. B.; de March, C. A.; van der Velden, W. J. C.; Ma, N.; Tewari, J.; del Torrent, C. L.; Li, L.; Faust, B.; Vaidehi, N.; Matsunami, H.; Manglik, A. Structural basis of odorant recognition by a human odorant receptor. *Nature* **2023**, *615*, 742–749.

(30) Hauser, A. S.; Kooistra, A. J.; Munk, C.; Heydenreich, F. M.; Veprintsev, D. B.; Bouvier, M.; Babu, M. M.; Gloriam, D. E. GPCR activation mechanisms across classes and macro/microscales. *Nature Structural & Molecular Biology* **2021**, *28*, 879–888.

(31) Lagerström, M. C.; Schiöth, H. B. Structural diversity of G protein-coupled receptors and significance for drug discovery. *Nature Reviews Drug Discovery* **2008**, *7*, 339–357.

(32) Venkatakrishnan, A. J.; Deupi, X.; Lebon, G.; Tate, C. G.; Schertler, G. F.; Babu, M. M. Molecular signatures of G-protein-coupled receptors. *Nature* **2013**, *494*, 185–194.

(33) Tehan, B. G.; Bortolato, A.; Blaney, F. E.; Weir, M. P.; Mason, J. S. Unifying family A GPCR theories of activation. *Pharmacology & Therapeutics* **2014**, *143*, 51–60.

(34) de March, C. A.; Yu, Y.; Ni, M. J.; Adipietro, K. A.; Matsunami, H.; Ma, M.; Golebiowski, J. Conserved residues control activation of mammalian G protein-coupled odorant receptors. *Journal of the American Chemical Society* **2015**, *137*, 8611–8616.

(35) Zarzycka, B.; Zaidi, S. A.; Roth, B. L.; Katritch, V. Harnessing ion-binding sites for GPCR pharmacology. *Pharmacological Reviews* **2019**, *71*, 571–595.
